# GC–MS hydrocarbon degradation profile data of *Pseudomonas frederiksbergensis* SI8, a bacterium capable of degrading aromatics at low temperatures

**DOI:** 10.1016/j.dib.2021.106864

**Published:** 2021-02-11

**Authors:** Oscar N. Ruiz, Osman Radwan, Richard C. Striebich

**Affiliations:** a Fuels and Energy Branch, Aerospace Systems Directorate, Air Force Research Laboratory, Wright-Patterson AFB, OH, USA; b Environmental Microbiology Group, University of Dayton Research Institute, Dayton, OH, USA; c Fuel Science Group, University of Dayton Research Institute, Dayton, OH, USA

**Keywords:** Psychrotrophs, *Pseudomonas frederiksbergensis*, Aromatics, Hydrocarbons, Jet fuel, GC–MS, Bioremediation, Biodeterioration, Biodegradation

## Abstract

The ability of the psychrotrophic bacterium *Pseudomonas frederiksbergensis* SI8 to grow and degrade aromatic hydrocarbons efficiently at low temperature is shown in this study. The robust growth of *P. frederiksbergensis* SI8 was demonstrated in jet fuel and an aromatic blend. The bacterium showed 2.5 to 3-fold faster growth in the aromatic blend than in jet fuel. The hydrocarbons degradation profile of *P. frederiksbergensis* SI8 at ambient temperature (i.e., 28 °C) and low temperature (i.e., 4 °C) was characterized by Gas Chromatography-Mass Spectrometry (GC–MS) analysis. GC–MS data demonstrated that *P. frederiksbergensis* SI8 is a novel psychrotrophic bacterium with the ability to degrade aromatic hydrocarbons at temperatures as low as 4 °C. Specifically, *P. frederiksbergensis* SI8 consumed toluene, ethylbenzene, n-propylbenzene and methyl ethyl benzene efficiently. The data presented here serves to characterize the hydrocarbon degradation profile of *P. frederiksbergensis* SI8 and corroborates the capacity of this bacterium to degrade aromatic hydrocarbons at low temperatures. The raw GC–MS data for the degradation of hydrocarbons by *P. frederiksbergensis* SI8 grown at 4 °C and 28 °C for 14 days have been deposited in Mendeley Data and can be retrieved from https://dx.doi.org/10.17632/z9292bvdmh.1 and https://dx.doi.org/10.17632/dp3sgwpj23.1. The datasets and raw data presented here were associated with the main research work “Metagenomic characterization reveals complex association of soil hydrocarbon-degrading bacteria” [1].

## Specifications Table

**Table:** 

Subject	Environmental Science
Specific subject area	Environmental Microbiology and Metagenomics
Type of data	Tables, Figures
How data were acquired	The hydrocarbon degradation profile of *Pseudomonas frederiksbergensis* SI8 was acquired and analysed using Gas Chromatography-Mass Spectrometry (GC–MS) in an Agilent 7890/5975 Gas Chromatographer-Mass Spectrometer.
Data format	Raw and Analysed
Parameters for data collection	*Pseudomonas frederiksbergensis* SI8 was grown at 4 °C and 28 °C for 14 days in 1 mL of M9 minimal medium overlaid with 10 µL of Jet A fuel.
Description of data collection	Hydrocarbon degradation profiles of bacteria grown in a mixture of M9 minimal medium with Jet A fuel as the carbon source were generated by GC–MS analysis.
Data source location	University of Dayton Research Institute**,** Dayton**,** Ohio**,** USA (39.7589, 84.1916).
Data accessibility	The tabulated raw data for Fig. 1, 2, 3, and Table 1 can be retrieved from https://dx.doi.org/10.17632/s3cfmcrbbs.2, https://dx.doi.org/10.17632/pszxfnhk2v.1, https://dx.doi.org/10.17632/zjzhmgs3vx.1 and https://dx.doi.org/10.17632/cvkbvx448x.1 Raw GC–MS hydrocarbon degradation data have been deposited in Mendeley Data and can be retrieved from https://dx.doi.org/10.17632/z9292bvdmh.1 and https://dx.doi.org/10.17632/dp3sgwpj23.1
Related research article	O. N. Ruiz, L. M. Brown, O. Radwan, L. L. Bowen, T. S. Gunasekera, S. S. Muller, Z. J. West, R. C. Striebich. Metagenomic Characterization Reveals Complex Association of Soil Hydrocarbon-Degrading Bacteria. International Biodeterioration & Biodegradation. **157**, 2021, 105,161, https://doi.org/10.1016/j.ibiod.2020.105161.

## Value of the Data

• Data from this work investigated the ability of *P. frederiksbergensis SI8* to grow and degrade selected specific aromatic hydrocarbons in fuel at low temperatures.
• *P. frederiksbergensis* SI8**,** a psychrotrophic bacterium, can be employed for bioremediation of aromatic hydrocarbons.
• Data generated by GC–MS can be used to better understand the microbial pathways and mechanisms for adaptation and biodegradation of fuel in cold environments.
• The rapid growth and efficient metabolization of toxic hydrocarbons by *P. frederiksbergensis* SI8 at room temperature and 4 °C is a rare trait in fuel-degrading microorganisms.
• The growth of the *P. frederiksbergensis* SI8 at low temperatures presents important implications for the biocontamination and biodeterioration of stored hydrocarbon fuels in cold weather environments.

## 1 Data Description

**Fig. 1:**
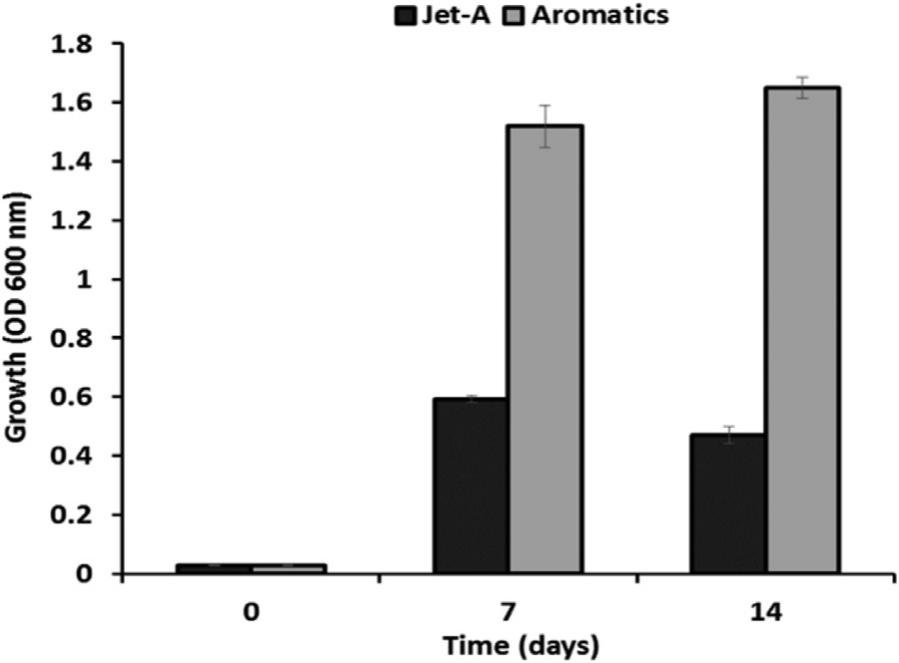
Growth of *Pseudomonas frederiksbergensis* SI8 in jet fuel and aromatic blend. *P. frederiksbergensis* SI8 was grown in M9 minimal medium in the presence of Jet A fuel and an aromatic blend (aromatics) for 14 days at 28 °C and its growth was determined by measuring OD_600_ at 0, 7 and 14 days. Tabulated raw data for this figure can be retrieved from https://dx.doi.org/10.17632/s3cfmcrbbs.2.

**Fig. 2:**
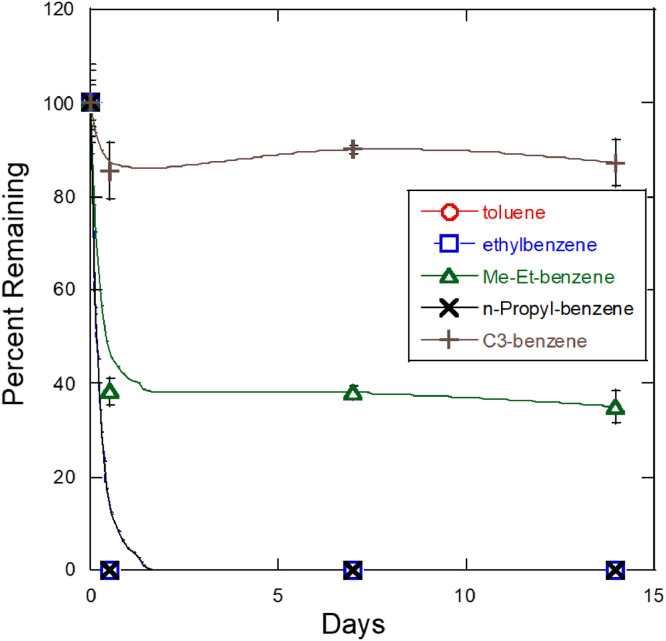
GC–MS profile of hydrocarbons degraded by *Pseudomonas frederiksbergensis* SI8 grown for 14 days at 28 °C in Jet A fuel. Tabulated raw data for this figure can be retrieved from https://dx.doi.org/10.17632/pszxfnhk2v.1.

**Fig. 3:**
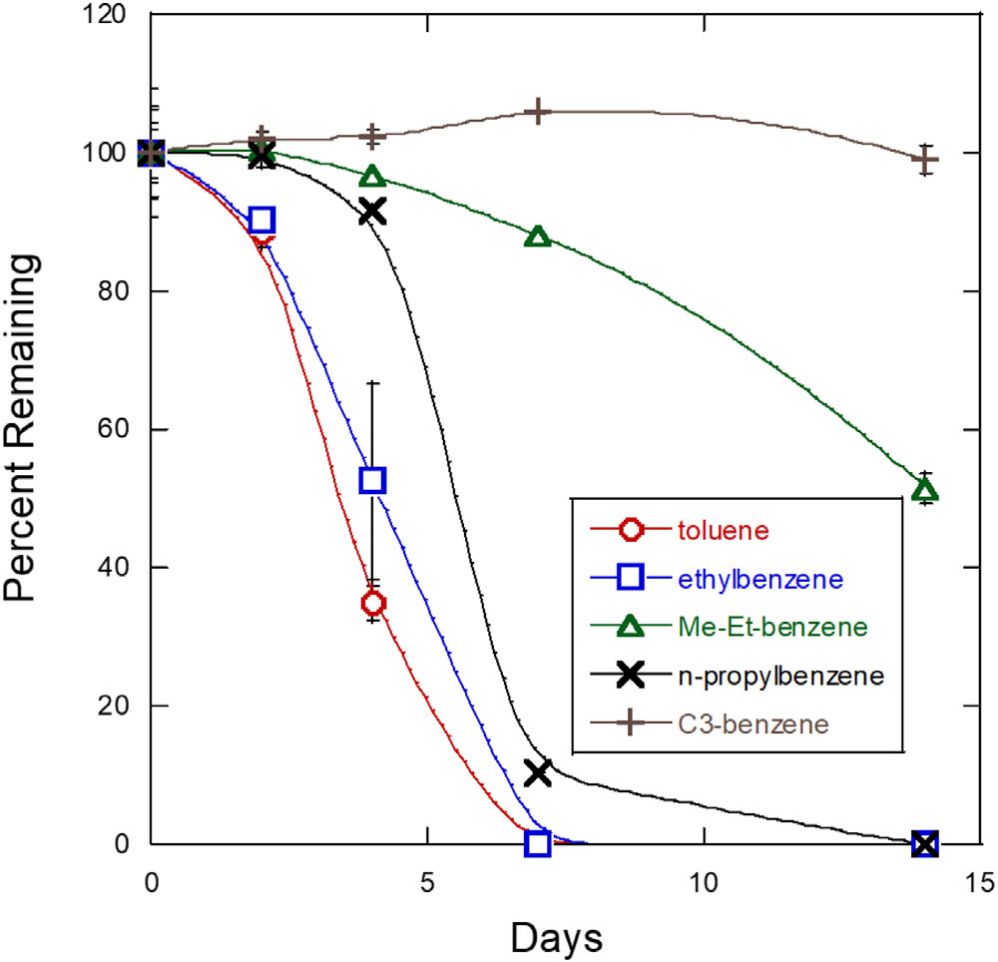
GC–MS profile of hydrocarbons degraded by *Pseudomonas frederiksbergensis* SI8 grown for 14 days at 4 °C in Jet A fuel. Tabulated raw data for this figure can be retrieved from https://dx.doi.org/10.17632/zjzhmgs3vx.1.

In this article, Fig. 1 provides the growth data comparison of *P. frederiksbergensis* SI8, a pychrotrophic soil bacterium isolated from a jet fuel enrichment [1] and identifed via genome sequencing [2], grown in M9 minimal medium amended with either Jet A fuel or a blend of aromatic hydrocarbons. The bacterial growth in the aromatic blend was 2.5-fold and 3-fold more at 7 and 14 days, respectively, than in Jet A fuel. Fig. 2 shows the GC–MS hydrocarbon degradation profile data demonstrating the ability of *P. frederiksbergensis* SI8 to proliferate and degrade hydrocarbons at 28 °C. Specifically, *P. fredericksbergensis* SI8 was observed to consume toluene, ethylbenzene, n-propylbenzene nearly to completion in only 0.5 day at room temperature. It also consumed other aromatic compounds at a lower rate, for example, methyl ethyl benzene which was degraded by about 40% from its original initial concentration in the fuel. Interestingly, other isomers of C3 alkyl benzene such as multi-ring aromatics (e.g., naphthalene) and cycloaromatics (e.g., indan, tetralin) were minimally degraded. While ethylbenzene was consumed immediately and completely, other 2-carbon substituted alkylbenzenes (o-, m- and p-xylene) were consumed poorly. Fig. 3 presents the GC–MS hydrocarbon degradation profile data demonstrating the ability of *P. frederiksbergensis* SI8 to proliferate and degrade hydrocarbons at 4 °C. The results showed that at 4 °C, it took 7 days to completely degrade the compounds that were previously degraded in just 0.5 days at 28 °C. *P. frederiksbergensis* SI8 degraded the same aromatic hydrocarbons at 28 °C and 4 °C (Figs. 2 and 3). Table 1, describes the degradation level of seventeen aromatic hydrocarbons present in Jet A fuel that was achieved by *P. frederiksbergensis* SI8 grown at 28 °C and 4 °C. The tabulated raw data for Figs. 1–3 and Table 1 can be retrieved from https://dx.doi.org/10.17632/s3cfmcrbbs.2,https://dx.doi.org/10.17632/pszxfnhk2v.1,https://dx.doi.org/10.17632/zjzhmgs3vx.1 and https://dx.doi.org/10.17632/cvkbvx448x.1, respectively.

**Table 1:** Degradation level for seventeen aromatic hydrocarbons after exposure of Jet A fuel to *P. frederiksbergensis* SI8 for 14 days at 4 °C and 28 °C. SD, standard deviation of *n* = 3. Tabulated raw data for this table can be retrieved from https://dx.doi.org/10.17632/cvkbvx448x.1.

	% Remaining, 14 days, *Pseudomonas frederiksbergensis* in Jet A			
Aromatic compound in Jet A	Average, 28 °C	SD (*n* = 3), 28 °C	Average, 4 °C	SD (*n* = 3), 4 °C
Toluene	0.0	0.0	0.0	0.0
Ethylbenzene	0.0	0.0	0.0	0.0
o-Xylene	80.8	3.8	97.3	1.3
Methyl-ethyl-benzene	34.9	3.5	51.5	2.2
n-Propyl-benzene	0.0	0.0	0.0	0.0
Ethyl-methyl-benzene-1	82.0	4.9	92.9	2.3
Ethyl-methyl-benzene-2	73.2	5.1	68.4	3.2
1,2,4-Trimethyl-benzene	87.2	4.9	99.1	2.0
Indane	80.4	6.0	84.9	2.6
n-Butyl-benzene	82.1	2.9	89.1	1.8
Ethyl-dimethyl-benzene-1	97.3	5.9	106.7	1.8
Ethyl-dimethyl-benzene-2	92.3	5.4	99.3	1.2
Methyl-indan	97.4	3.0	104.7	5.9
Naphthalene	87.3	6.1	90.3	2.1
2-Methyl-naphthalene	97.3	4.8	99.0	1.2
1-Methyl-naphthalene	97.1	4.7	98.9	2.1
Dimethyl-naphthalene	101.4	3.1	98.8	1.5

## 2 Experimental Design, Materials and Methods

### 2.1 Measuring bacterial growth in Jet A fuel and aromatic blend

*P. frederiksbergensis* SI8 was grown overnight in Tryptone Soy Broth (TSB) at 28 °C with agitation at 200 RPM. The bacterial cells were harvested by centrifugation at 11,000 RPM for 10 min at room temperature, washed three times with M9 minimal medium. The bacterial pellet was resuspended in M9 and concentration measured spectrophotometrically at OD_600_. A final concentration of 0.03 OD_600_ was adjusted in the aqueous phase of a mixture of 15 mL of M9 minimal medium overlaid with 15 mL of either Jet A fuel or aromatic blend. The bacterial culture was incubated for 14 days at 28 °C with agitation at 200 RPM and the bacterial growth was measured using a spectrophotometer at OD_600_.

### 2.2 Hydrocarbon degradation assay for GC–MS profiling

*P. frederiksbergensis* SI8 culture was prepared following the above procedure and added at a concentration of 0.01 OD_600_ to 1 mL of M9 minimal medium overlaid with 10 μL of Jet A fuel in an 8 mL glass vial [1]. Negative control vials contain M9 minimal medium overlaid with 10 μL of Jet A fuel without bacterial inoculation were used. The three replicate vials for each of the condition and time point were airtight sealed using a Teflon-lined lid to prevent hydrocarbon volatilization, and finally were incubated at 4 °C and 28 °C for the length of the experiment. Triplicate samples along with negative control were prepared for GC–MS analysis at 5, 10 and 15 days.

### 2.3 Sample extraction for GC–MS analysis

Both samples and negative control were prepared for GC–MS analysis as previously described [1,3]. Briefly, an extraction of the hydrocarbons in each vial was performed using HPLC grade hexanes [1,3] with a ratio of 1 (Jet A) to 200 (hexanes) followed by mixing. The hexanes layer was quantitatively recovered and used in GC–MS analysis.

### 2.4 GC–MS analysis

The extracted hexane with the fuel hydrocarbons was analyzed using an Agilent 7890/5975 Gas Chromatographer-Mass Spectrometer using the split-injection method. To elute 1 μL sample for MS analysis, a non-polar 30 m column (DB5-MS, Agilent Technologies) with helium carrier gas in the full scan mode column was heated from 40 °C to 280 °C at a rate of 5 °C per minute. The MS scanning range was *m/z* 33–350 and extracted ions were used to quantify the signal. Signals from exposed samples were compared to the negative control samples to determine the remaining concentration of hydrocarbon compounds as a function of time. The final concentration of each compound, in both sample and negative control, was normalized to farnesane (a non-degradable hydrocarbon) as an internal standard. Experiments were conducted in triplicate and the results plotted as a function of time.

## CRediT Author Statement

**Oscar N Ruiz**: Conceptualization, Supervision, Investigation, Writing - Original draft preparation, Reviewing and Editing; **Osman Radwan:** Visualization, Data curation, Writing - Original draft preparation; **Richard C. Striebich**: Investigation, Methodology, Visualization, Data curation, Writing - Original draft preparation.

## Declaration of Competing Interest

The authors declare that they have no known competing financial interests or personal relationships that could have appeared to influence the work reported in this paper.
